# Child and Adolescent Behavior Inventory (CABI): A New Instrument for Epidemiological Studies and Pre-Clinical Evaluation

**DOI:** 10.2174/1745017901915010044

**Published:** 2019-02-28

**Authors:** Carlo Cianchetti, Andrea Pittau, Valeria Carta, Grazia Campus, Roberta Littarru, Maria Giuseppina Ledda, Alessandro Zuddas, Giuseppina Sannio Fancello

**Affiliations:** 1 Clinic of Child and Adolescent Neuropsychiatry, University Azienda Ospedaliero-Universitaria, Cagliari, Italy; 2 Gnosis Center for Neuropsychological and Emotional Evaluation, Cagliari, Italy

## Child and Adolescent Behavior Inventory (CABI): A New Instrument for Epidemiological Studies and Pre-Clinical Evaluation

Clinical Practice & Epidemiology in Mental Health, **2013**, 9: 51-61

**Correction:**

Few corrections have been provided and replaced online in 15^th^, 20^th^, 21^st^ and 22^nd^ rows of the Appendix. 

**Original:**

**Table:** C.A.B.I. QUESTIONNAIRE FOR PARENTS By Carlo Cianchetti M.D., University of Cagliari, Italy

Name of child or youth:______________________________________________________________________ Sex: M□ F□ Date of birth:_______/_______/_____ Age:__________ Class:_________ Date of compilation:_____/____/_____ Compiler: mother (name)___________________________________father (name)__________________________________________

**Instructions**: The following statements refer to problems which may be present in children/youth. Please answer as regards your child and what has taken place during the last six months. For each statement, ask yourself if the situation is **very true, somewhat or sometimes true, **or **not true. **Answer by marking an “X” in the appropriate square. Some questions may not apply to your son or daughter if he/she is very young, as the questionnaire also regards adolescents; however, please answer all the questions. If the meaning of one or more questions in unclear to you, or you are unable to answer, immediately note the number of the question/s at the bottom of the questionnaire and when you hand it in, ask for explanations.

**Table:** 

		**Very True**	**Somewhat or** **Sometimes True**	**Not True**
1	Your son/daughter often complains about some physical discomfort (for example: a headache, stomach ache, *etc.*)	□	□	□
2	He is excessively worried about illnesses and/or that he will get ill	□	□	□
3	He finds it difficult to fall asleep or says he does not sleep well	□	□	□
4	His sleep is disturbed by nightmares or waking up during the night	□	□	□
5	He appears tense and/or anxious	□	□	□
6	He tends to worry about everything	□	□	□
7	He worries about school too much	□	□	□
8	It is hard for him to be separated or far from his parents	□	□	□
9	He is excessively shy	□	□	□
10	He is usually embarrassed around strangers or people he does not know very well	□	□	□
11	He is excessively afraid of something (e.g. the dark, being alone, insects, thieves) Specify what he is afraid of	□	□	□
12	He is excessively afraid of dirt, so he has to wash continually	□	□	□
13	There are repetitive actions or “rituals” that he frequently repeats and he says he cannot help doing them, If yes, describe which ones	□	□	□
14	He has an obsessive need for things to be in a precise order	□	□	□
15	He is vepleasant thoughts and cannot free himself from them	□	□	□
16	He is very afraid of making mistakes	□	□	□
17	It is hard for him to make decisions, even about unimportant things	□	□	□
18	Has he ever been involved in or witnessed particularly stressful events, after which his behaviour changed in some way? If true, indicate what behavioural changes occurred after the event	□	□	□
19	He cries for no reason or about unimportant things	□	□	□
20	He is often in a black mood (“depressed”)	□	□	□
21	He says or shows that he is not happy	□	□	□
22	He shows no interest, not even in pleasant things	□	□	□
23	He shows no interest, not even in pleasant thibgs	□	□	□
24	He feels inferior to others; he has low self-esteem	□	□	□
25	He is often tired or listless; everything exhausts him	□	□	□
26	He blames himself too much	□	□	□
27	He has sometimes said he does not want to live any longer	□	□	□
28	He has hurt himself or tried to hurt himself	□	□	□
29	He is very irritable	□	□	□
30	He often gets angry, even about unimportant things	□	□	□
31	He has frequent mood changes	□	□	□
32	He is quick-tempered and has fits of anger	□	□	□
33	He does not obey and it is difficult to make him obey	□	□	□
34	He does not follow the rules	□	□	□
35	He often tells lies or cheats	□	□	□
36	He is domineering and always wants to assert himself	□	□	□
37	He quarrels frequently	□	□	□
38	He bothers and intentionally annoys others	□	□	□
39	He often hits people	□	□	□
40	He destroys things	□	□	□
41	He is or has been cruel to animals or people	□	□	□
42	He has committed petty theft	□	□	□
43	He is impulsive and acts before thinking	□	□	□
44	He tends not to take turns when he is playing	□	□	□
45	He interrupts, disturbing games and others’ conversations	□	□	□
46	He is always moving around and cannot stay still	□	□	□
47	He cannot sit down for a long time but has to get up	□	□	□
48	He runs and jumps everywhere in an exaggerated way	□	□	□
49	He has trouble concentrating while doing his homework	□	□	□
50	He has trouble paying attention to something for a long period	□	□	□
51	He gets tired very quickly even when he is playing	□	□	□
52	He feels persecuted	□	□	□
53	He is overly suspicious	□	□	□
54	Sometimes he has strange ideas	□	□	□
55	Sometimes he says he sees or hears things that are not there	□	□	□
56	He has difficulty in relating to and interacting with others	□	□	□
57	He cannot make real friends or does not seem interested in doing so	□	□	□
58	He does not play willingly with his peers	□	□	□
59	He does not seem to express emotions using appropriate facial expressions	□	□	□
60	His behaviour is "strange", unlike that of his peers	□	□	□
61	He asks inappropriate questions, like overly-personal questions to strangers at inopportune times	□	□	□
62	He sometimes wets the bed	□	□	□
63	He sometimes dirties his pants during the day	□	□	□
64	He stuffs himself with food	□	□	□
65	He keeps to a strict diet (not prescribed by a doctor or dietician)	□	□	□
66	He feels too fat or says that parts of his body are too fat	□	□	□
67	He has recently lost a lot of weight	□	□	□
68	He appears to be overly interested in sex	□	□	□
69	He shows he would like to be of the opposite sex	□	□	□
70	He smokes	□	□	□
71	He drinks alcohol	□	□	□
72	He uses drugs (smokes hashish or other dangerous substances)	□	□	□
73	He does not do well at school	□	□	□
74	He has recently done much worse at school	□	□	□
75	His classmates or other children make fun of him, threaten or mistreat him	□	□	□

**Corrected:**

**Table:** C.A.B.I. QUESTIONNAIRE FOR PARENTS By Carlo Cianchetti M.D., University of Cagliari, Italy

Name of child or youth:______________________________________________________________________ Sex: M□ F□ Date of birth:_______/_______/_____ Age:__________ Class:_________ Date of compilation:_____/____/_____ Compiler: mother (name)___________________________________father (name)__________________________________________

**Instructions**: The following statements refer to problems which may be present in children/youth. Please answer as regards your child and what has taken place during the last six months. For each statement, ask yourself if the situation is **very true, somewhat or sometimes true, **or **not true. **Answer by marking an “X” in the appropriate square. Some questions may not apply to your son or daughter if he/she is very young, as the questionnaire also regards adolescents; however, please answer all the questions. If the meaning of one or more questions in unclear to you, or you are unable to answer, immediately note the number of the question/s at the bottom of the questionnaire and when you hand it in, ask for explanations.

**Table:** 

		**Very True**	**Somewhat or** **Sometimes True**	**Not True**
1	Your son/daughter often complains about some physical discomfort (for example: a headache, stomach ache, *etc.*)	□	□	□
2	He is excessively worried about illnesses and/or that he will get ill	□	□	□
3	He finds it difficult to fall asleep or says he does not sleep well	□	□	□
4	His sleep is disturbed by nightmares or waking up during the night	□	□	□
5	He appears tense and/or anxious	□	□	□
6	He tends to worry about everything	□	□	□
7	He worries about school too much	□	□	□
8	It is hard for him to be separated or far from his parents	□	□	□
9	He is excessively shy	□	□	□
10	He is usually embarrassed around strangers or people he does not know very well	□	□	□
11	He is excessively afraid of something (e.g. the dark, being alone, insects, thieves) Specify what he is afraid of	□	□	□
12	He is excessively afraid of dirt, so he has to wash continually	□	□	□
13	There are repetitive actions or “rituals” that he frequently repeats and he says he cannot help doing them, If yes, describe which ones	□	□	□
14	He has an obsessive need for things to be in a precise order	□	□	□
15	He is obsessed by unpleasant thoughts and cannot free himself from them	□	□	□
16	He is very afraid of making mistakes	□	□	□
17	It is hard for him to make decisions, even about unimportant things	□	□	□
18	Has he ever been involved in or witnessed particularly stressful events, after which his behaviour changed in some way? If true, indicate what behavioural changes occurred after the event	□	□	□
19	He cries for no reason or about unimportant things	□	□	□
20	He often seems sad	□	□	□
21	He is often in a black mood (“depressed”)	□	□	□
22	He says or shows that he is not happy	□	□	□
23	He shows no interest, not even in pleasant things	□	□	□
24	He feels inferior to others; he has low self-esteem	□	□	□
25	He is often tired or listless; everything exhausts him	□	□	□
26	He blames himself too much	□	□	□
27	He has sometimes said he does not want to live any longer	□	□	□
28	He has hurt himself or tried to hurt himself	□	□	□
29	He is very irritable	□	□	□
30	He often gets angry, even about unimportant things	□	□	□
31	He has frequent mood changes	□	□	□
32	He is quick-tempered and has fits of anger	□	□	□
33	He does not obey and it is difficult to make him obey	□	□	□
34	He does not follow the rules	□	□	□
35	He often tells lies or cheats	□	□	□
36	He is domineering and always wants to assert himself	□	□	□
37	He quarrels frequently	□	□	□
38	He bothers and intentionally annoys others	□	□	□
39	He often hits people	□	□	□
40	He destroys things	□	□	□
41	He is or has been cruel to animals or people	□	□	□
42	He has committed petty theft	□	□	□
43	He is impulsive and acts before thinking	□	□	□
44	He tends not to take turns when he is playing	□	□	□
45	He interrupts, disturbing games or others’ conversations	□	□	□
46	He is always moving around and cannot stay still	□	□	□
47	He cannot sit down for a long time but has to get up	□	□	□
48	He runs and jumps everywhere in an exaggerated way	□	□	□
49	He has trouble concentrating while doing his homework	□	□	□
50	He has trouble paying attention to something for a long period	□	□	□
51	He gets tired very quickly even when he is playing	□	□	□
52	He feels persecuted	□	□	□
53	He is overly suspicious	□	□	□
54	Sometimes he has strange ideas	□	□	□
55	Sometimes he says he sees or hears things that are not there	□	□	□
56	He has difficulty in relating to and interacting with others	□	□	□
57	He cannot make real friends or does not seem interested in doing so	□	□	□
58	He does not play willingly with his peers	□	□	□
59	He does not seem to express emotions using appropriate facial expressions	□	□	□
60	His behaviour is "strange", unlike that of his peers	□	□	□
61	He asks inappropriate questions, like overly-personal questions to strangers at inopportune times	□	□	□
62	He sometimes wets the bed	□	□	□
63	He sometimes dirties his pants during the day	□	□	□
64	He stuffs himself with food	□	□	□
65	He keeps to a strict diet (not prescribed by a doctor or dietician)	□	□	□
66	He feels too fat or says that parts of his body are too fat	□	□	□
67	He has recently lost a lot of weight	□	□	□
68	He appears to be overly interested in sex	□	□	□
69	He shows he would like to be of the opposite sex	□	□	□
70	He smokes	□	□	□
71	He drinks alcohol	□	□	□
72	He uses drugs (smokes hashish or other dangerous substances)	□	□	□
73	He does not do well at school	□	□	□
74	He has recently done much worse at school	□	□	□
75	His classmates or other children make fun of him, threaten or mistreat him	□	□	□

